# Structure of personal disorders in hypertensive disease patients

**DOI:** 10.1192/j.eurpsy.2021.677

**Published:** 2021-08-13

**Authors:** N. Chernus, S. .Sivkov, A. Serdakova, A. Sivkov, T. Savina

**Affiliations:** 1 Department Of Outpatient Therapy, I.M. Sechenov First Moscow State Medical University: Moscow, Russia, Moscow, Russian Federation; 2 Phd In Medicine, Associate Professor Of The Department Of Clinical Pharmacology And Internal Diseases Propaedeutics, the I.M. Sechenov First Moscow State Medical University, Moscow, Russian Federation; 3 Department Of Psychology, the I.M. Sechenov First Moscow State Medical University, Moscow, Russian Federation; 4 The Department Of Clinical Pharmacology And Internal Diseases Propaedeutics, the I.M. Sechenov First Moscow State Medical University, Moscow, Russian Federation

**Keywords:** hypertensive disease, anxiety, anger, depression

## Abstract

**Introduction:**

The strcuture of personal disorders in hypertensive disease aptients remains relevant topic.

**Objectives:**

The study population included 57 hypertensive disease patients; mean age 49,1+9,6 years old (42 females and 15 males). The control group included 62 healthy individuals (49 females and 13 males); mean age 48,1+8,6 years old.

**Methods:**

Emotional condition of subjects was assessed using the Depression Scale of Zung, the Spielberger trait Anger scale and Anxiety, the Toronto Alexithymia Scale and SCL – 90-R Questionnaire.

**Results:**

The study results showed that as compared to the healthy individuals, the hypertensive disease patients showed significantly higher scores of reactive anxiety (46,0+9,0 and 39,0+8,2; р<0,01), personal anxiety (50,3+9,2 and 41,03+7,9; р<0,01), depression (42,7+7,2 and 36,59+5,95;р<0,01), alexithymia (69,4+8,8 and 59,0+9,2; р<0,01), state anger (11,8+3,6 and 10,6+1,8; р<0,01), reactive anger (9,2+2,6 and 8,1+2,4; р<0,05), personal anger (21,4+5,3 and 18,1+4,6; р<0,01), trait anger (8,3+3,0 and 7,3+2,3; р<0,05), self-aggression (16,2+4,9 and 13,4+3,8; р<0,01), aggression towards others (15,9+3,9 and 14,7+3,4; р<0,05), somatization (1,27+0,6 and 0,5+0,4; р<0,01), hostility (1,2+0,7 and 0,5+0,4; р<0,01), obsessive-compulsive traits (1,2+0,7 и 0,6+0,4; р<0,01), psychoticism (0,7+0,6 and 0,27+0,30; р<0,01) and paranoid traits (1,22+0,6 and 0,5+0,4; р<0,01), phobic anxiety (0,6+0,5 and 0,2+0,2; р<0,01) and interpersonal sensitivity (1,2+0,7 and 0,7+0,5; р<0,01).

**Conclusions:**

Close interrelations between manifestations of anxiety and depression spectrum disorders and anger may be explained by internal conflict between aggressive impulses and the need for adaptive behavior in such individuals, resulting in consistent vicious vortex.

